# Supplementary material for: Content-rich biological network constructed by mining PubMed abstracts
Source: BMC Bioinformatics. 2004 Oct 8;5:147. doi: 10.1186/1471-2105-5-147 (PMC528731; doi:10.1186/1471-2105-5-147)
Supplement: Additional File 2 — The original results of the above study (non-essential files are deleted to keep the file size under the limit set by BMC bioinformatics). [file 1471-2105-5-147-S2.bz2 › chilibotAdditionalFile2/dip05/309ID10393925E1338/html/PSEN1_FLNA.html]

 


 **PSEN1** and **FLNA** 
  
Found 3 abstracts in PubMed, retrieved 3.  
 

 What does Google say? 
 PDF only 
| .edu only 

---

**Interactive relationship** (e.g. stimulation, inhibition, etc)

**Neutral relationship**- Overexpression of PS1  [ **PSEN1** ]  appears to modify the distribution of ABP280  [ **FLNA** ]  and Fh1 proteins in cultured cells.  Ref: 9437013 J Neurosci, 1998
- We report here that the loop regions of PS1  [ **PSEN1** ]  and PS2 interact with nonmuscle filamin  [ **FLNA** ]  actin binding protein 280  [ **FLNA** ] , ABP280  [ **FLNA** ]  and a structurally related protein filamin homolog 1, Fh1.  Ref: 9437013 J Neurosci, 1998

**Non-interactive relationship** (e.g. studied together, co-existance, homology, etc.)

- Detection of ABP280  [ **FLNA** ]  Fh1 proteins in these structures suggests that these presenilin  [ **PSEN1** ]  interacting proteins may be involved in the development of AD.  Ref: 9437013 J Neurosci, 1998
- Cell surface PS1  [ **PSEN1** ]  formed complexes in vivo with actin binding protein filamin ABP 280  [ **FLNA** ] , which is known to form bridges between cell surface receptors and cytoskeleton and mediate cell adhesion and cell motility.  Ref: 10393925 Proc Natl Acad Sci U S A, 1999
